# Acupuncture analgesia involves modulation of pain-induced gamma oscillations and cortical network connectivity

**DOI:** 10.1038/s41598-017-13633-4

**Published:** 2017-11-24

**Authors:** Michael Hauck, Sven Schröder, Gesa Meyer-Hamme, Jürgen Lorenz, Sunja Friedrichs, Guido Nolte, Christian Gerloff, Andreas K. Engel

**Affiliations:** 1 Department of Neurophysiology and Pathophysiology, University Medical Center Hamburg-Eppendorf, 20246 Hamburg, Germany; 2 Department of Neurology, University Medical Center Hamburg-Eppendorf, 20246 Hamburg, Germany; 3 HanseMerkur Center for Traditional Chinese Medicine at the University Medical Center Hamburg-Eppendorf, 20246 Hamburg, Germany; 4 Faculty of Life Science, Laboratory of Human Biology and Physiology, Applied Science University, 21033 Hamburg, Germany

## Abstract

Recent studies support the view that cortical sensory, limbic and executive networks and the autonomic nervous system might interact in distinct manners under the influence of acupuncture to modulate pain. We performed a double-blind crossover design study to investigate subjective ratings, EEG and ECG following experimental laser pain under the influence of sham and verum acupuncture in 26 healthy volunteers. We analyzed neuronal oscillations and inter-regional coherence in the gamma band of 128-channel-EEG recordings as well as heart rate variability (HRV) on two experimental days. Pain ratings and pain-induced gamma oscillations together with vagally-mediated power in the high-frequency bandwidth (vmHF) of HRV decreased significantly stronger during verum than sham acupuncture. Gamma oscillations were localized in the prefrontal cortex (PFC), mid-cingulate cortex (MCC), primary somatosensory cortex and insula. Reductions of pain ratings and vmHF-power were significantly correlated with increase of connectivity between the insula and MCC. In contrast, connectivity between left and right PFC and between PFC and insula correlated positively with vmHF-power without a relationship to acupuncture analgesia. Overall, these findings highlight the influence of the insula in integrating activity in limbic-saliency networks with vagally mediated homeostatic control to mediate antinociception under the influence of acupuncture.

## Introduction

A network of multiple cortical areas underlies the multidimensional sensory and emotional experience of pain^1^. Cortical areas involved in pain processing include the primary (SI) and secondary somatosensory cortex (SII), limbic areas such as the insula and the cingulate gyrus, midbrain and brainstem areas and the prefrontal cortex (PFC). Somatosensory regions are widely accepted to represent sensory-discriminative aspects whereas limbic areas seem to be more important for emotional and motivational aspects of pain processing^2^. Numerous studies demonstrated that gamma-band synchronization between different brain areas allows binding of stimulus features into a coherent percept^3–6^. Furthermore, evidence exists that changes in thalamo-cortical oscillatory synchrony in the gamma range result in more efficient stimulus processing^4,7–9^. Gamma-band oscillations in somatosensory and limbic areas can also be observed following experimental pain stimuli^10–12^. Pain-related brain areas are not solely activated by noxious stimuli^13^ but also engaged by other emotional and cognitive processes. Hence, gamma-band synchronization across different cortical areas may indicate how the brain orchestrates the various sensory, cognitive and emotional components of pain perception. Furthermore, pain-induced gamma-band activity can be modulated by top-down and bottom-up attention leading to altered pain perception^11,14,15^. Thus, gamma-band oscillations are likely to reflect both the integration of activity across different pain-related regions underlying subjective pain experience and its endogenous modulation by attention and expectation.

Although the specificity and mechanisms of acupuncture effects are under debate^16^ some evidence is available suggesting an involvement of different processes at all levels of pain processing including peripheral tissue reactions, modulation of spinal cord processes, as well as subcortical and cortical modulations. For example, local transmitter release has been made responsible for the acupuncture effect in the periphery^17,18^. Central mechanisms include a modulation of µ-opioid receptors which has been observed in cingulate cortex, caudate, insula, thalamus and amygdala^19^, presumably representing a similar mechanism as in placebo analgesia. Some authors interpret acupuncture as stress-related analgesia^20^. Furthermore, evidence is available that acupuncture can modulate the autonomic nervous system (ANS)^21–25^. Recent studies support the view that acupuncture might modulate pain through the interaction of cortical sensory, limbic and executive networks with longer lasting changes in the default mode network and the ANS^26^.

To investigate the influence of pain modulation by acupuncture on pain related communication processes and responses of the ANS we tested the influence of sham and verum acupuncture in a double-blind repeated measures design on cortico-cortical coupling and heart rate variability (HRV) following experimental pain induced by laser radiant heat stimuli. On two different experimental days, subjects were either treated with a verum or sham acupuncture. ECG and EEG were recorded and further analyzed with a focus on HRV analysis as well as power and coupling of neural gamma oscillations. We expected acupuncture analgesia to be associated, on the one hand, with changes of inter-regional coherence of gamma-oscillations between pain-relevant brain structures and, on the other hand, with changes in the sympatho-vagal balance as derived from HRV.

## Results

### Behavioral data

After completion of recordings on the second experimental day, the subjects were told that only one session day involved a verum acupuncture, the other day a sham acupuncture. Subsequently, we asked them to estimate in percent (100% = totally certain and −100% totally uncertain) about how certain they would be to judge on which day they received sham or verum treatment. The mean correct estimation rate was 23,1%, but did not reach significance based on a one-sample t-test (t_(25)_ = 1.8; p = 0.078). Therefore, subjects were not systematically able to distinguish between sham and verum acupuncture. Subjects who received sham treatment on the first day estimated with 41,7% correctly and subjects who received acupuncture on the first day estimated 7,1% correctly the treatment order. However, no significant difference between the groups in estimation error was found (t_(25)_ = 1.4; p = 0.175). Furthermore, subjects were asked, whether they believe in the effectiveness of acupuncture in general and whether it would work also for themselves. Furthermore, the subjects were asked, if they consider themselves as religious. 84 ± 16% believed that acupuncture is effective in general and 78 ± 19% believed it would also work for themselves. The two belief variables correlated significantly (r = 0.7; p < 0.01). 40 ± 35% of the subjects declared themselves religious, which was unrelated to their belief in effectiveness of acupuncture. Furthermore, we divided all subjects in religious and non-religious subjects and compared the groups, which showed no significant differences in belief on effectiveness of acupuncture.

In the beginning and the end of each experimental day, we performed a pain-threshold estimation. On the sham day, the mean laser-pain threshold was 295 ± 66 mJ before and 419 ± 39 mJ after the experiment. On the acupuncture day, the mean laser-pain threshold was 275 ± 90 mJ before and 431 ± 93 mJ after the experiment. The repeated 2 × 2 ANOVA with the factors treatment and repetition showed a significant effect for repetition (F_(25)_ = 110; p = <0.001) and no significant effect of treatment on the laser-pain threshold estimation.

**Figure 1:**
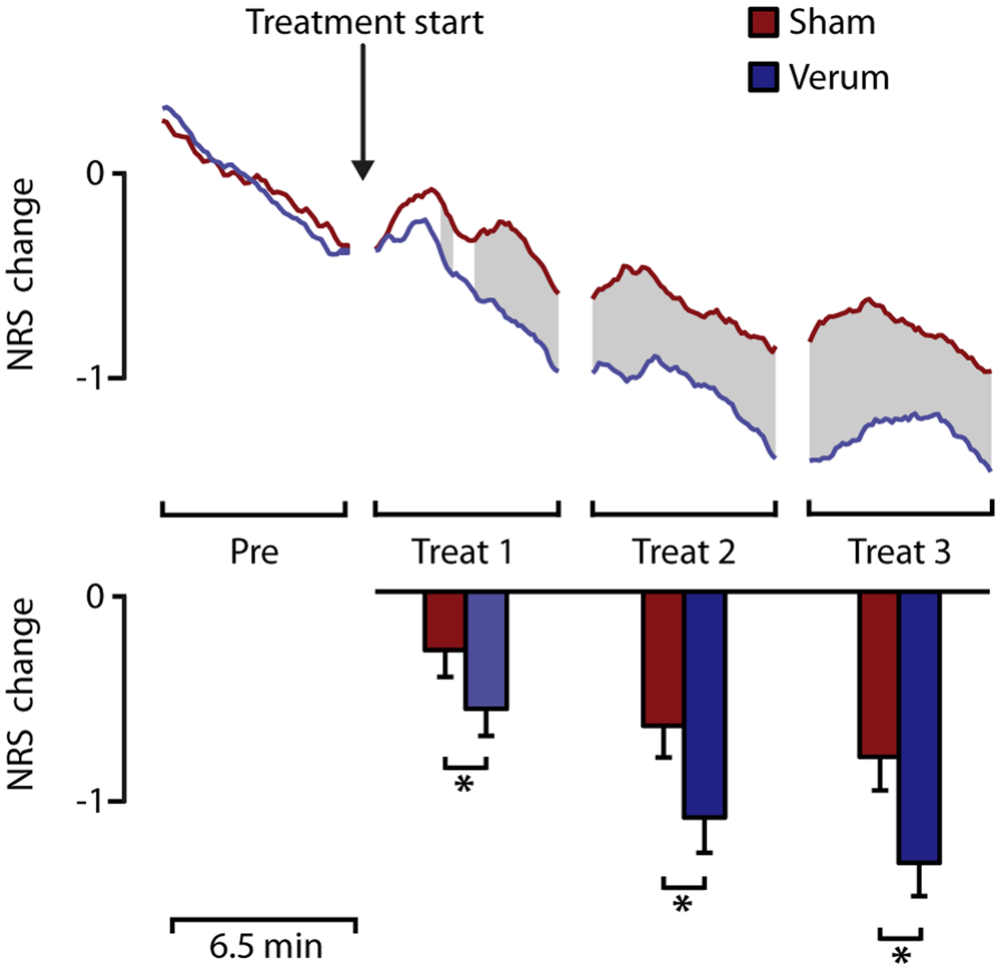
Pain ratings. The top panel shows the grand mean time course of pain-rating change (NRS change). All pain ratings were normalized to the mean pain rating value of the pre-block. Significant differences based on a running t-test are shaded in grey. The bottom panel shows the mean NRS change for each block. The asterisks highlight significant differences based on a paired t-test. Besides a significant effect with lower pain ratings after successive block repetition, acupuncture treatment induced a stronger reduction of pain ratings compared to placebo treatment in each treatment block.

The pain ratings were normalized to the mean values of the pre-block and subsequent numerical rating scale (NRS) changes were calculated. All NRS change values were normal distributed based on a Kolmogorov-Smirnov Test. The time course of NRS-change in pain-ratings and the mean values of each block repetition and treatment are shown in Fig. 1.

Based on a running t-test with a cluster randomization approach to correct for multiple comparisons, significant differences between sham and verum acupuncture started during the first treatment block. Furthermore, a 2 × 4 ANOVA with the factors treatment and block repetition was calculated for the mean pain-ratings of each block. Significant main effects were found for the variable treatment (F_(1,24)_ = 11.6; p < 0.01) and repetition (F_(3,72)_ = 36.2; p < 0.001). We also found a significant treatment by repetition interaction (F_(3,72)_ = 6.6; p < 0.01). Subsequent block wise paired t-tests were calculated to evaluate the main effect treatment. During treatment-block 1 the pain-rating change was significantly lower (t_(25)_ = 2.4; p < 0.05) for verum treatment (−0.6 ± 0.6 NRS-change) compared to sham treatment (−0.3 ± 0.6 NRS-change). During treatment-block 2 the pain-rating change was significantly lower (t_(25)_ = 3.4; p < 0.05) for acupuncture treatment (−0.6 ± 0.7 NRS-change) compared to sham treatment (−1.0 ± 0.8 NRS-change). During treatment-block 3 the pain-rating change was significantly lower (t_(25)_ = 3.3; p < 0.05) for verum treatment (−0.8 ± 0.8 NRS-change) compared to sham treatment (−1.2 ± 0.9 NRS-change).

### Heart rate variability

**Figure 2:**
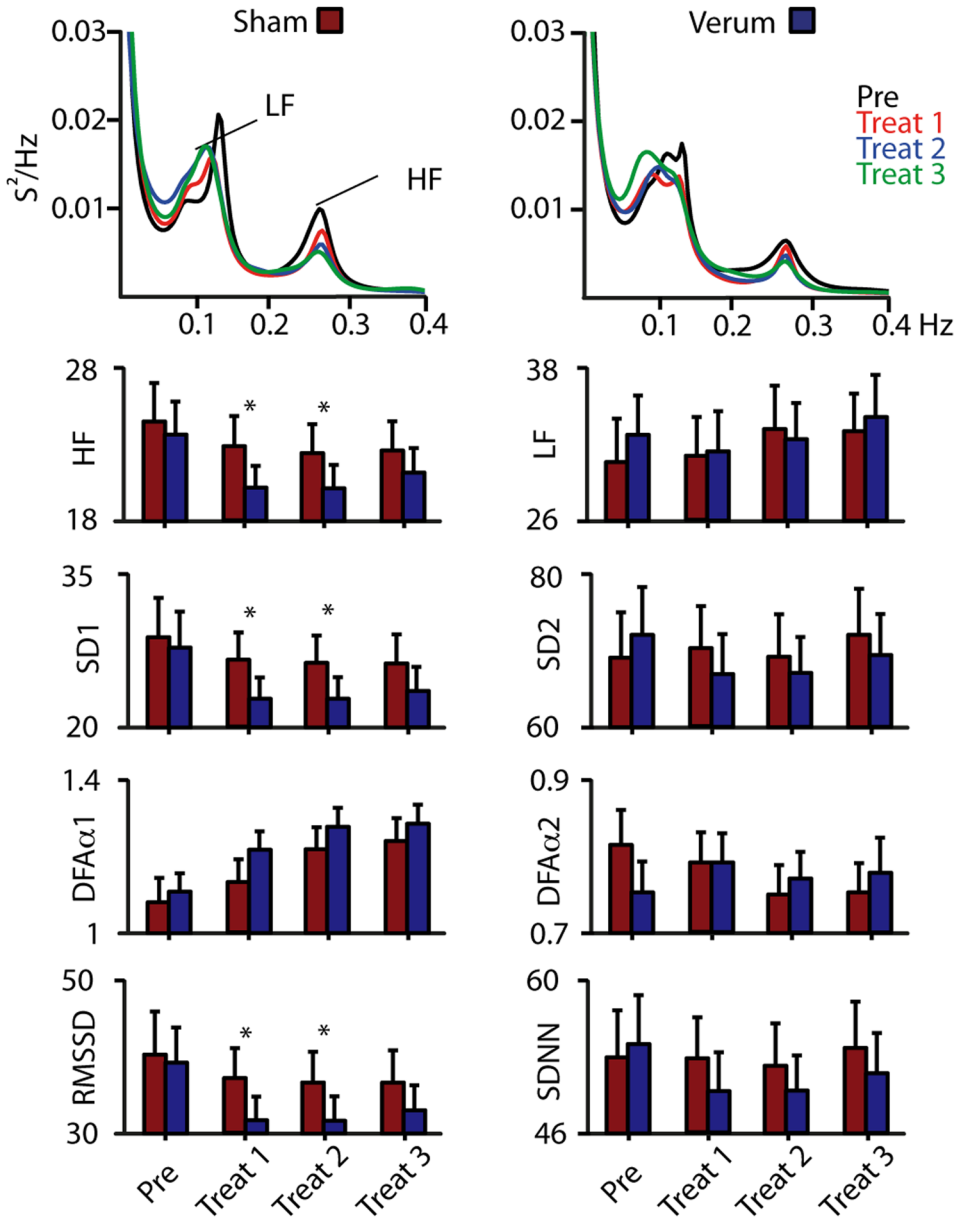
Heart rate variability. Top panel: ECG spectra for sham and verum condition revealed LF (0.04–0.15 Hz) and HF (0.15–0.4 Hz) peaks. Short-term parameters such as the HF-Spectrum, SD1, DFA-alpha-1 and RMSSD indicate a decrease in vagal activity over time for both conditions. This effect was significant stronger, using a paired t-test, during verum treatment for treatment block 1 and 2 as indicated by asterisks. Long-term parameters such as the LF-spectrum, SD2, DFA-alpha-2 and SDNN were unaffected by repetition and treatment manipulation.

Analysis of HRV focused on the evaluation of short and long-term parameters as indication of a change in the sympatho-vagal balance under the effects of verum and sham acupuncture. Results are shown in Fig. 2. All described HRV parameters were fed into a 2 × 4 ANOVA with the factors treatment and block repetition. Mean R-R-intervals did not change over all study parameters as did not all long-term HRV parameters (SDNN, LF-power, SD2 and DFA-alpha-2). Only short-term HRV parameters (RMSSD, HF-power, SD1 and DFA-alpha-1) showed significant effects for the factors treatment and block repetition.

For the HF-power, we found a significant main effect for the factor acupuncture treatment (F_(1,24)_ = 5.1; p < 0.05, Greenhouse-Geisser corrected) and block-repetition (F_(3,72)_ = 3.7; p < 0.05, Greenhouse-Geisser corrected). Further paired t-tests revealed that the verum acupuncture induced less HF-power during treatment block 1 (t_(25)_ = 2.8; p < 0.05) and block 2 (t_(25)_ = 2.8; p < 0.01) compared to the sham condition. Furthermore, treatment block 1 (t_(25)_ = 3.0; p < 0.01) and treatment block 2 (t_(25)_ = 3.1; p < 0.01) had significantly smaller HF-power compared to the pre block (Fig. 2) in the verum condition only.

The ANOVA regarding SD1 values showed a marginally significant effects of condition (F_(1,24)_ = 3.9; p = 0.06, Greenhouse-Geisser corrected) and repetition (F_(1,24)_ = 3.3; p = 0.06, Greenhouse-Geisser corrected). Paired t-test of the SD1 data revealed significantly lower SD1 values for the verum condition during treatment block 1 (t_(25)_ = 2.3; p < 0.05) and treatment block 2 (t_(25)_ = 2.4; p < 0.05). Further t-test revealed lower SD1 values of treatment block 2 (t_(25)_ = −4.7; p < 0.001) and treatment block 3 (t_(25)_ = −4.3; p < 0.001) for the sham condition compared to the pre block. For the verum treatment all post treatment blocks showed significantly lower SD1 values compared to the pre block (treatment block 1 vs pre: t_(25)_ = −3.1; p < 0.05; treatment block 2 vs pre: t_(25)_ = 4.9; p < 0.001; treatment block 3 vs pre: t_(25)_ = −4.3; p < 0.001).

The ANOVA regarding DFA-alpha-1 values revealed a significant repetition effect (F_(3,72)_ = 22.0; p < 0.001, Greenhouse-Geisser corrected). The RMSSD values showed a marginal condition (F_(1,24)_ = 3.9; p = 0.06, Greenhouse-Geisser corrected) and repetition (F_(1,24)_ = 3.4; p = 0.06, Greenhouse-Geisser corrected) effect. Further paired t-tests revealed that the verum acupuncture induced lower RMSSD during treatment block 1 (t_(25)_ = 2.3; p < 0.05) and block 2 (t_(25)_ = 2.5; p < 0.05) compared to the sham condition.

Taken together, all predominantly vagally mediated short-term HRV parameters (RMSSD, HF-power, SD1 and DFA-alpha-1) decreased during verum acupuncture. All long-term HRV parameters (SDNN, LF-power, SD2, DFA-alpha-2) did not change significantly. The LF power slightly increased leading to an increase of the LF/HF ratio over time, which indicates a shift towards stronger influence of the sympathetic nervous system during acupuncture. This effect was positively correlated with a decrease in pain ratings in the verum treatment.

### Gamma oscillations

**Figure 3:**
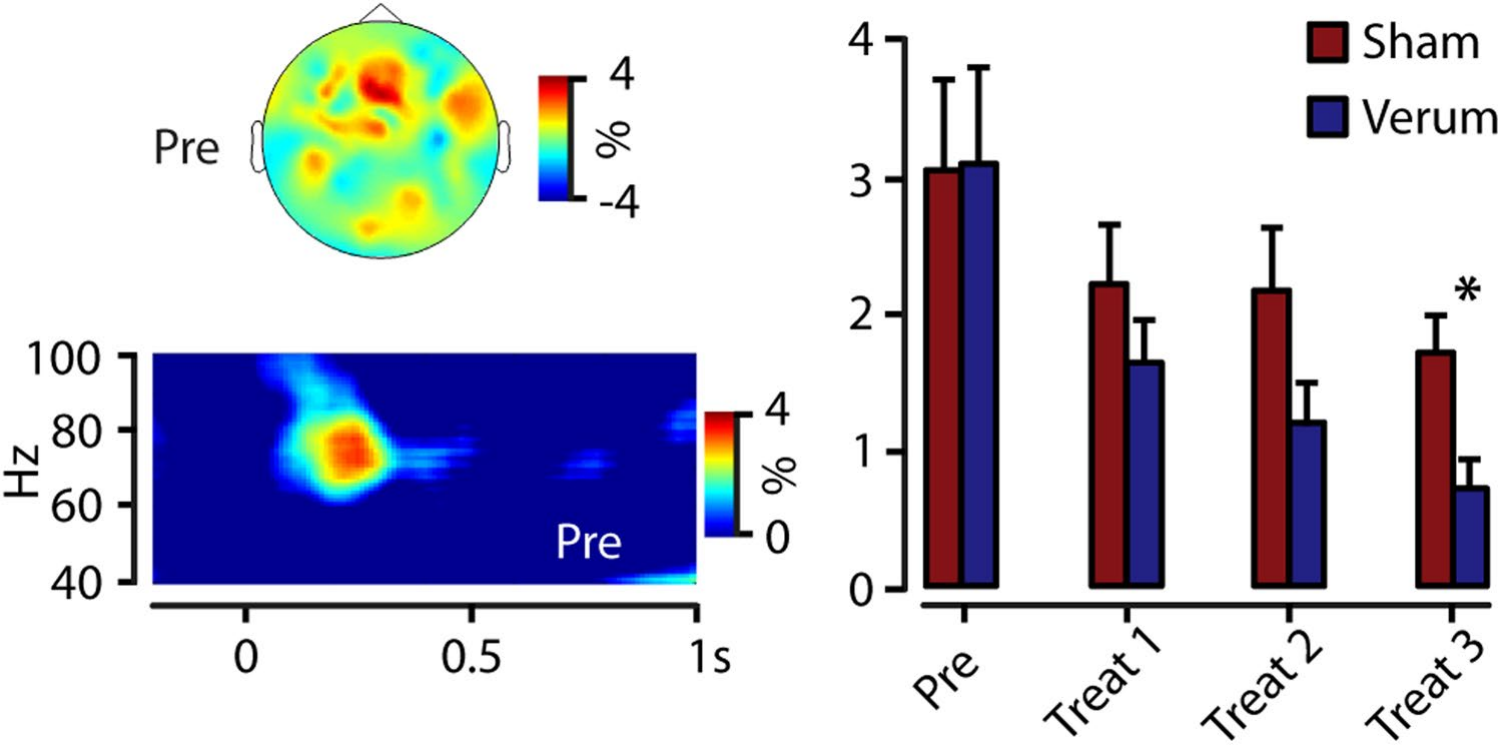
Gamma oscillations. Top left: Our analysis revealed laser-pain induced gamma oscillations with a centro-frontal topographical distribution. Bottom Left: Time-frequency analysis of signals within this centro-frontal ROI showed a maximum at 74 Hz and 240ms for the grand mean data. This gamma activity decreased over time and the gamma decrease was significantly stronger, based on paired t-tests, during the last treatment block for verum acupuncture compared to the sham condition (right panel), as indicated by an asterisk.

The grand average of total power across subjects revealed a laser-pain induced gamma-power increase (maximum 74 Hz, 240 ms), with a centro-frontal topographical distribution (Fig. 3). Power values of a central ROI were fed into a 2 × 4 ANOVA with the factors treatment and block repetition. Significant main effects were found for the variable treatment (F_(1,24)_ = 4.3; p < 0.05) and repetition (F_(3,72)_ = 8.4; p < 0.01). Subsequent block-wise paired t-tests were calculated to evaluate the main effect of treatment. The difference between sham and verum treatment was only significant (t_(25)_ = 3.0; p < 0.05) in the last experimental group with lower gamma power in the verum condition (1.9 ± 1.3%) compared to the sham condition (1.1 ± 0.9%).

To evaluate potential influences of changes the baseline interval on pain-induced oscillations and to investigate systematic differences of vigilance-depended resting state oscillations we analyzed the alpha-power (8–14 Hz) in the baseline period (−1000 to 0ms). Power values of an occipital ROI were fed into a 2 × 4 ANOVA with the factors treatment and block repetition. No significant main effects were found for the variable treatment (F_(1,24)_ = 0.5; p < 0.85) or for the variable repetition (F_(3,72)_ = 2.3; p < 0.08).

**Figure 4:**
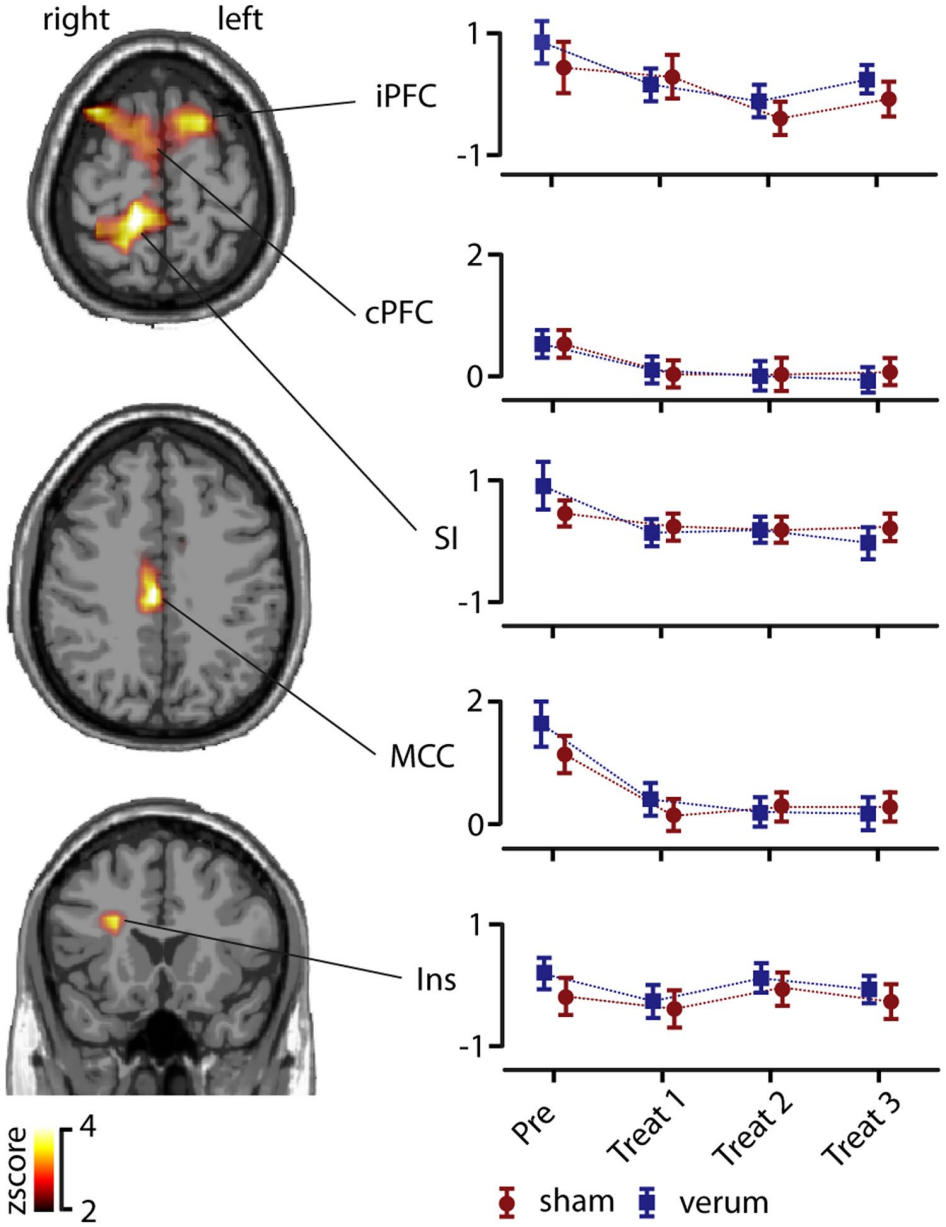
Source localization of pain-induced gamma power. Using eLORETA five significant gamma-activation clusters were localized in the contralateral insula (Ins), mid-cingulate cortex (MCC), contralateral primary somatosensory cortex (SI) and prefrontal cortex (PFC). Colour-scaled Z-scores based on a cluster randomization approach are projected on a MNI-standard brain. Power analysis within these anatomical locations revealed a significant repetition effect (based on a two-way ANOVA) within MCC and SI. No significant power effect was found between sham and verum treatment on the source level.

In order to investigate regional specificity of the above-described gamma modulation we applied a distributed source reconstruction technique termed eLORETA (Pascual-Marqui 2007) to the data. Statistical maps represented by *z* scores were generated by comparison between baseline and post stimulus condition (see materials and methods) using the peak activation cluster from the power analysis. After a cluster randomization approach, five significant sources were identified within the gamma range (Fig. 4). Dipole strength obtained within each cluster was further analyzed to investigate treatment and repetition effects. First a contralateral activation in the insula (Talairach coordinates X = 44; Y = −11; Z = 17) was identified. The 2 × 4 ANOVA of the dipole strength revealed no significant main effects nor interactions. Second, a contralateral activation in the primary somatosensory cortex (SI) was identified (X = 30; Y = −25; Z = 62), which showed a significant main effect for repetition (F_(1)_ = 3.2; p < 0.05). Further paired t-tests revealed that only the first block had a significant higher dipole strength compared to the repetition block 2 (t_(25)_ = 2.4; p < 0.05), block 3 (t_(25)_ = 2.3; p < 0.05) and block 4 (t_(25)_ = 2.3; p < 0.05). Furthermore, we were able to identify an activation cluster within the mid cingulate cortex (MCC; X = 2; Y = −12; Z = 31). Results of the 2 × 4 ANOVA showed a significant main effect for repetition (F_(1,24)_ = 13.2; p < 0.01). Further paired t-tests revealed that only the first block had a significant higher dipole strength compared to the repetition block 2 (t_(25)_ = 4.1; p < 0.01), block 3 (t_(25)_ = 4.1; p < 0.01) and block 4 (t_(25)_ = 4.9; p < 0.01). Another activation for the gamma band was found in bilateral dorsolateral prefrontal cortices (PFC; X = −22; Y = 28; Z = 42 and X = 30; Y = 28; Z = 39). The 2 × 4 ANOVA of the dipole strength did not revealed significant main effects or interactions. Taken together, the power of gamma oscillations at the source level was modulated in contralateral insula, contralateral SI, MCC and bilateral PFC. Only activation in SI and the MCC was affected by the experimental repetition manipulation. However, no differences in oscillatory power between sham or verum treatment were found on the source level (Fig. 4).

### Connectivity analysis

**Figure 5:**
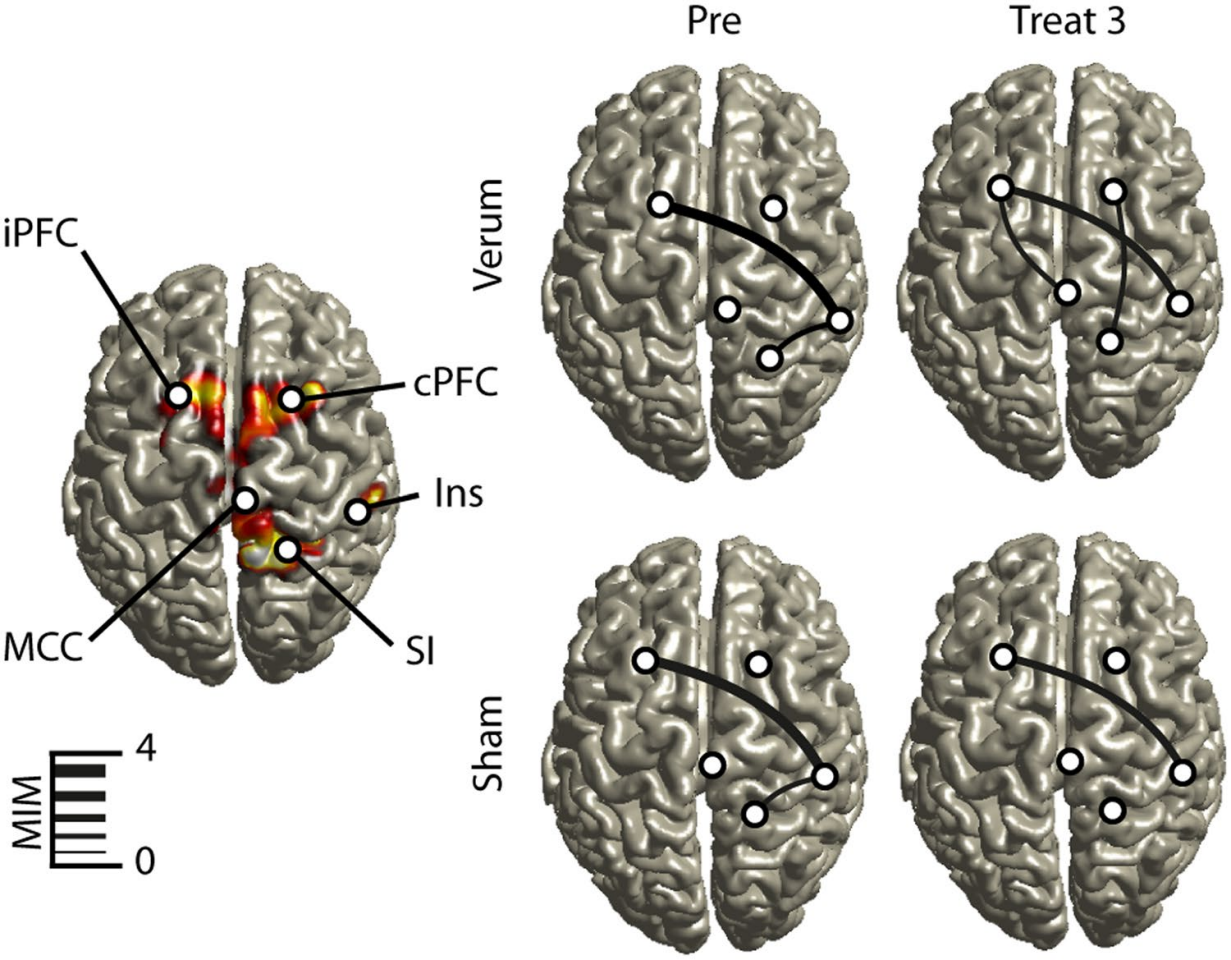
Coupling of gamma oscillations. Using the multivariate interaction measure (MIM), neuronal coupling was analyzed between the five regions obtained from source reconstruction results, as shown on the left panel. Only statistically significant connections (based on paired t-tests using z-normalization of MIM values) are shown and the strength of connectivity is expressed by the thickness of the line. The top row show data for verum treatment, the bottom row those for sham treatment. Before the intervention (Pre) connections were identical for both days. Comparing the last block (Treat 3), more connections were present in the verum condition between both prefrontal cortices (PFC) and mid cingulate cortex (MCC), insula (Ins) and primary somatosensory cortex (SI).

To analyze coupling of gamma-band oscillations in source space we used the imaginary coherence-based multivariate interaction measure (MIM). MIM is a measure to investigate synchronization processes of brain sources in the EEG/MEG, which are robust to artifacts of volume conduction. This can be achieved by maximization of imaginary coherency in virtual channels on the source level (see methods). Based on the source localisation of gamma oscillations, five regions of interest were chosen for connectivity analysis, namely bilateral PFC, MCC, contralateral SI and contralateral insula. To reveal significant functional connections, differences in pain-induced MIM were calculated between pre- and post-stimulus intervals (Fig. 5). During the pre-block, we were able to identify two significant connections. One significant connection was found between ipsilateral PFC and contralateral insula. The second significant connection was found between the contralateral insula and contralateral SI. Interestingly, these connections were the same on both experimental days during the pre-block, indicating a strong retest reliability (Fig. 5). During both treatment days, the connections between the ipsilateral PFC and the contralateral insula remained stable but with decreasing MIM values over time. Furthermore, during verum treatment, connection between ipsilateral PFC and contralateral MCC as well as contralateral PFC and contralateral SI were significant in the treatment block 3 at the end of the experiment. These connections were not present during sham stimulation in the treatment block 3. Furthermore, the connection between contralateral PFC and SI was not only present during verum treatment, but also in the treatment block 1 in the sham treatment condition. Taken together, during verum treatment there is evidence for increased neuronal communication in the gamma band between prefrontal sides and the cingulate gyrus, insula and SI.

**Figure 6:**
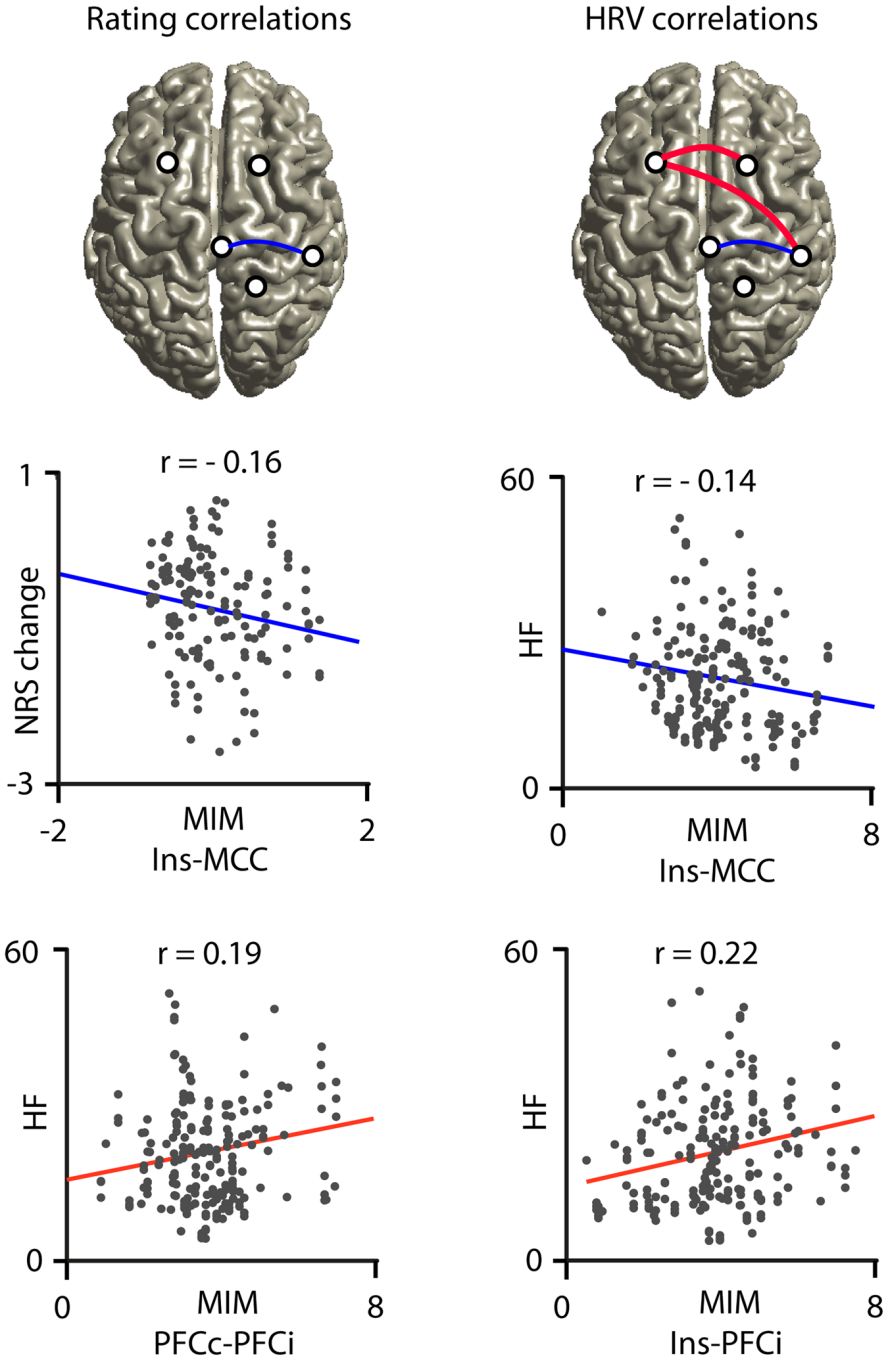
Correlation between cortical connectivity, pain ratings and HRV parameters. Change in pain ratings (NRS change) was significant negatively correlated (blue line) with the strength of pain induced coupling (multivariate interaction measure, MIM) between contralateral insula (Ins) and mid-cingulate cortex (MCC). The same negative correlation was found between MIM and the HF spectral power value (HF). Furthermore, we identified positive correlations (red lines) between HF parameters and MIM for connections between insula and ipsilateral prefrontal cortex (PFCi) and between both prefrontal cortices (PFCc and PFCi).

To evaluate relationships between connectivity measures, pain ratings and HRV parameters a correlation analysis was performed using pain induced MIM parameters together with pain ratings and HRV parameters (Fig. 6). For this analysis parameters were pooled over both conditions. Changes in pain ratings were significantly negatively correlated (r = −0.16, p < 0.05) with MIM parameters between insula and MCC. The same connection was negatively correlated with the HF-spectrum values (r = −0.14, p < 0.05). This indicates that the higher the connectivity between insula and MCC the greater is the reduction of both pain and vmHF-power. This is compatible with the assumption that an increase in neuronal communication between insular and cingulate parts of the limbic network mediate decreased pain during the experiment together with decreased vagal tone. On the other hand, neuronal coupling between left and right PFC (r = 0.19, p < 0.01) as well as between ipsilateral PFC and insula (r = 0.22, p < 0.01) was positively correlated with an increase in HF power. This finding could indicate that with stronger inter-hemispheric connectivity within prefrontal cortex and with increased prefrontal-insular connectivity the vagal tone is less weakened. Overall, these findings highlight the influence of the insula in connection with hubs of cognitive-executive and limbic-saliency networks to mediate antinociception and HRV-parameters under the exteroceptive processing of verum, but also to some degree sham acupuncture.

## Discussion

We examined the analgesic effects of verum in comparison to sham acupuncture on laser-induced pain using a double-blind controlled study design in healthy participants. Our analysis focused on the modulation of laser-induced gamma oscillations in pain-related brain regions and on changes of inter-regional coupling in this frequency band by acuptuncture. Verum treatment diminished pain more than sham treatment. Furthermore, we observed a decrease of vagally mediated HRV components (vmHRV) in both treatments, which again was significantly stronger in the verum than in the sham acupuncture group. This effect indicates a predominant negative modulation of the vagal influence on HRV by acupuncture. The major drive for the vmHRV comes from the baroreceptor input via bronchial, diaphragmatic and aortic afferent activity to the nucleus tractus solitarii due to breathing-related changes in intrathoracic pressure, giving rise to respiratory sinus arrhythmia^27^. A baroreceptor receptor loop over the nucleus tractus solitarii and the rostroventral medulla to the sinus node also mediates a sympatho-excitatory slow HRV component. One of the reasons why vagal and sympathetic outflow of the autonomic nervous system differentially influence the respective rapid and slow HRV components is that postganglionic autonomic efferents have an about tenfold slower conduction velocity than preganglionic fibers. Whereas parasympathetic efferents have longer preganglionic rapidly conducting pathways with ganglia near the sinus node, sympathetic efferents synapse already in the stellate ganglia close to the cervical vertebral column and reach the sinus node over significantly longer slowly conducting postganglionic pathways. Yet, given the antagonistic interaction of vagal and sympathetic activity within a central autonomic network in the brainstem, the negative modulation of vmHRV components by acupuncture can be the result of a combination of parasympatho-inhibitory and sympatho-excitatory effects. However, both parasympatho-inhibitory and sympatho-excitatory effects on the heart can also be modulated indirectly through pathways linking the frontal cortex with the autonomic network^27^. It is therefore also possible that an acupuncture-mediated change in frontal networks may have caused this shift in sympatho-vagal balance associated with reduction of pain sensitivity, an explanation that we want to further elaborate by discussing the results of the EEG analysis.

Pain-related gamma oscillations in the EEG were more strongly decreased during verum than sham treatment in our study. These gamma oscillations were localized in bilateral PFC, SI, MCC and the insula. Connectivity analysis between these regions showed a relation of coupling between insula and MCC to both pain sensitivity and a reduction of vmHRV. Stronger coupling of insula with PFC or between left and right PFC resulted in greater vmHRV, but left pain sensitivity unchanged. It has been suggested^26^ that vmHRV indicates a central state in which interoception and activation of the default mode network (DMN) predominate in the cortex. States with increased processing of exteroceptive input, especially from salient stimuli such as pain or other task-relevant stimuli, are believed to give rise to a dominance of strongly task-related networks like the saliency network or the central executive network, which are anti-correlated to the DMN. Given the pivotal role of both MCC and insula in the saliency network, it is therefore possible that acupuncture as exteroceptive stimulus shifts brain network activity from DMN towards task-related networks. However, our paradigm did not allow to analyse coupling within the DMN because we only studied gamma oscillations induced by laser pain stimuli but did not have sufficient recording periods of ongoing activity. Thus, we can only speculate that stronger decrease of DMN activity might have occurred as effect of verum compared to sham acupuncture, which indirectly could be reflected in the stronger reduction of vmHRV. This view is consistent with that of Bai *et al*.^26^ that acupuncture might modulate pain through a shift in the interaction of different cortical sensory, limbic and executive networks with the DMN and the autonomic nervous system^26^.

It is possible that other effects might have contributed to the observed reduction of pain sensitivity and vmHRV in our study. Habituation effects due to repetitive stimuli^28^ or shifts in attentional mechanisms could have decreased perceived pain in our study. The subjects were told that the experimental purpose was to test the reliability and effectiveness of acupuncture. Interestingly, most of the subjects expressed a general belief in the effectiveness of acupuncture and the belief that acupuncture would also be effective on themselves. Therefore, it is possible that both conditions, namely sham and verum treatment induced an expectation of analgesia equivalent to placebo cognitions that could have contributed to the reduced painfulness of laser stimuli. For this study, we chose a sophisticated double blind randomized study design, the effectiveness of which has been already approved in clinical trials^25,29^. However, even though not statistically significant, 23% of the subjects recognized the treatment order correctly, which could have biased verum acupuncture towards greater placebo cognitions than sham treatment. The belief in the effectiveness of a treatment is a strong pain modulator^30^ not only in placebo experiments. If placebo effects in fact influenced verum acupuncture more than sham acupuncture in our study we cannot exclude that placebo effects may have reduced pain and vmHRV more in the verum condition. Furthermore, expectation-related placebo analgesia involves a fronto-cingulate descending pathway, which projects to the periaqueductal grey^31^ and engages the endogenous opioid system^32^. Sympatho-excitatory outflow and descending pain inhibition pathways converge anatomically in the rostroventral medulla. Therefore, it remains an important question whether placebo and acupuncture share common pathways to mediate analgesia. Acupuncture per se can alter autonomic function as well. Most experiments, which investigated autonomic and HRV parameters were performed without the presence of pain. In these studies, divergent results are reported, which may be due to the different study designs and different approaches of acupuncture^22^. Taken together, it is likely that acupuncture modulates the autonomic nervous system and, when associated with painful stimulation, contributes to a decrease in parasympathetic activity.

In summary, we were able to show that pain induced gamma oscillations in pain-related brain areas are more strongly reduced by verum than sham acupuncture. Furthermore, vagally-mediated high-frequency ECG power and other short-term HRV-parameters derived from time-domain and non-linear analysis decreased also more strongly during verum than sham acupuncture treatment. Reduction of pain ratings and vmHRV parameters were significantly correlated with increase of connectivity between the insula and the mid-cingulate cortex. Overall, these findings suggest that the insula may play a key role in regulating the interplay between limbic-saliency networks and homeostatic autonomic control to mediate antinociception under the influence of exteroceptive signals triggered by acupuncture.

## Methods

### Participants

Thirty-two healthy right handed participants were recruited and 26 subjects completed the study (19 female), aged 20 to 29 years (mean 25.3). Participants received monetary compensation. Six subjects were discarded or dropped out from the study due to strong artifacts in the EEG recordings (n = 3) such as strong movement and muscle artefacts, strong habituation to laser stimuli sensation after the threshold estimation (n = 2) or an unusually low pain threshold (n = 1), although basic neurological investigation did not reveal any abnormalities. Exclusion criteria were claustrophobia, neurological disorders, MR-incompatibilities and positive drug or alcohol history. Subjects were informed that they could terminate the experiment at any time and written informed consent was obtained form all participants. The study was conducted in accordance with the Declaration of Helsinki and was approved by the ethic committee of the medical association Hamburg (Ethik-Kommision der Ärztekammer Hamburg, Study number PV4732).

### Pain stimulus, procedure and pain rating

We delivered brief infrared laser stimuli of 1 ms duration and a beam diameter of 5 mm to an 4 cm^2^ area at the left elbow using a Thulium YAG laser (wavelength 2 μm, StarMedTec, Starnberg, Germany). The stimulation side was in a circular area (radius 2 cm) close to acupoint *LI11 (Quchi). T*he laser was slightly shifted within this area after each stimulus to avoid skin habituation and sensitisation effects. Prior to the experiment participants were familiarized with the use of a 11-point numerical pain rating scale ranging from 0 (no sensation) to 10 (maximal pain). On this rating scale, a value of 0 indicates no sensation at all and 3 indicates the threshold for a pain sensation. Sensation higher than 0 and below 3 indicates non-painful warm, barely tactile sensations, whereas sensation at pain threshold indicates the beginning of a painful hot and stinging pain. Values of 10 denote worst imaginable pain. Individual pain threshold was tested by calculating the average intensity at which subjects reported first a rating value above 3 in three ascending stimulus series and, moreover, first a rating value below 3 in three descending series of laser stimuli using successive intensity increments of 20 mJ^33^. Pain thresholds were collected before and after each experimental day. During the experiment subjects were comfortably seated in an electrically shielded and sound-attenuated recording chamber with their eyes closed. Each block consisted of 60 laser pain stimuli with an intensity at two-fold of the individual pain threshold. The inter-stimulus interval varied between 6 or 7 seconds. Three seconds after the laser stimulus, an acoustic event (2000-Hz tone) prompted a verbal response using the numerical rating scale (NRS, see above) as already used for threshold testing. In total, each subject participated in two experimental days with 4 experimental blocks, which were identical for verum and sham treatments. The experiment started with a pre-block and 60 laser stimuli. This block was followed by the treatment intervention (see below). After the treatment, three further blocks with 60 laser stimuli were applied in identical manner (block 1 –block 3).

### Treatment intervention

We conducted two identical experimental blocks on two non-consecutive days in a counterbalanced double-blind design. Participants were informed that they were invited to test the reliability and effectiveness of acupuncture. Sham acupuncture was performed on one day and verum acupuncture on the other day in a random order. All participants as well as the investigator and the acupuncturist were blinded regarding the type of treatment. This procedure was only possible by using special acupuncture needles, the so-called press-tack needles (Pyonex; Seirin Corporation, Shizuoka, Japan). These small needles have a diameter of 0.2 mm and length of 0.6 mm and are fixed on an adhesive round plaster. The sham needles are visually identical to the verum needles with the needle element removed from the shaft. Due to the small size of the needles, the application usually does not induce any sensation and verum needles cannot be distinguished from sham devices and can be removed from the puncture site without obvious skin alteration^29^. Verum and sham plasters were similarly applied on distant extremities other than the site of laser stimuli. The treatment concept has been developed based on the traditional theory of reflex areas on distant corresponding body regions^34^. The pattern of treatment was selected by a systematic physical examination for pain sensitive reflex areas (Ashi) immediately beforehand the application of sham or verum plasters^35^. This acupuncture protocol has been successfully approved in a randomized double-blind study on adhesive capsulitis^36^. Following the pre-block laser stimulation at the left radial elbow, several verum or sham plasters were affixed in pain sensitive areas to the contralateral right elbow, the contralateral right knee and the ipsilateral left knee. The experimental procedure can be seen in supplementary Figure 1. The placements of sham and verum plasters distant from the experimental laser-pain side were chosen to ensure a minimization of peripheral counter irritation effects. Two acupuncturists with more than 10 years of clinical experience and versed in the acupuncture protocol method performed the plaster positioning. Three study nurses were involved in the experimental arrangement in order to ensure a double-blind treatment procedure.

### Randomisation and blinding

The order of verum and sham treatment was allocated to the volunteers by study nurse 1 according to a randomization procedure with a 1:1 allocation ratio. Random numbers were previously generated and assigned serially to the participants by an independent researcher using Microsoft Office Excel 2007. Assignment codes were concealed into identical opaque envelopes and the envelopes were sealed. Prior to a participant´s first treatment, study nurse 1 opened the assigned envelope after she had written the participant’s name on it and prepared a tray containing needles according to his/her group assignment. All trays looked alike; verum and sham needles were placed in such a way that others could not identify them as verum or sham. Before treatment, study nurse 2 obtained the tray; the study nurses did not communicate with each other. Study nurse 2 delivered the tray to the acupuncturist and supervised the treatment procedure. In this way, they both witnessed each other’s and the participant’s complying with the study protocol and prevented each other from examining the needles. Upon completion of the experiment study nurse 3 removed the needles. She was not allowed to examine them or to communicate neither with the participant nor any person involved in the study. Removed press tacks were disposed of in a punctured-resistant, rigid container for biomedical waste, whose content cannot be accessed at all.

### Heart rate variability

The ECG was recorded simultaneously with the EEG and the same amplifier settings were used (see below). Electrodes were placed in the fourth intercostal space (Wilson V1) and the left anterior axillary line (Wilson V5). All heart rate variability analysis was performed using the Kubios®-HRV software (http://kubios.uef.fi)^37^ together with Matlab. For each subject and experimental block, time-based, frequency-based and nonlinear parameters were calculated. Detection of the QRS-complex results in the so-called normal-to-normal intervals (NN) and QRS detection was visually controlled for each subject and condition. Time based parameters included RMSSD (square root of the mean squared differences of successive NN intervals) and SDNN (standard deviation of the NN intervals). Frequency based parameters were estimated using autoregressive modeling with a model order of 32. Frequency estimates were computed separately for low frequency (LF, 0.04–0.15 Hz) and high frequency power (HF, 0.15–0.4) according to the guidelines of the European Society of Cardiology. Before statistical testing the square root was taken of the LF and HF band power. Non-parametrical parameters included DFA-alpha-1, DFA-alpha-2 (detrended fluctuation analysis), SD1 and SD2 (Poincaré plot derived geometrical parameters). Time-based, frequency-based and non-parametrical ECG results can be divided into short-term parameters (RMSSD, HF, SD1 and DFA-alpha-1) and long-term parameters (SDNN, LF, SD2 and DFA-alpha-2). Short-term parameters reflect rapid changes of R-R intervals, which are mediated by the parasympathetic nervous system via vagal innervation of the sinus node^38,39^. Long term parameters or slow fluctuations of R-R intervals are primarily modulated by sympathetic, but also parasympathetic influences^40^.

### Data acquisition and analysis of EEG

The EEG was recorded using 128 channels (including 2 EOG-channels and 2 ECG channels, EASYCAP) and BrainVision Recorder software (Brain Products GmbH, Gilching, Germany) through four BrainAmp MRplus 32 channel amplifiers with a sampling frequency of 1000 Hz and a band pass filter between 0.1–250 Hz. The electrode impedance was kept below 15 kΩ. The EEG was recorded with nose reference. The data were analyzed offline using fieldtrip (www.ru.nl/fcdonders/fieldtrip), freely available open source toolboxes running under Matlab (The Mathworks, Natick, MA) and custom made Matlab code. Firstly, data were band-pass-filtered from 0.3 to 100 Hz and down-sampled to 400 Hz. Then the continuous data sets were epoched in segments from −1000 to 3000 ms relative to pain stimulus onset. Artifact removal was done by visual inspection of all segments for the presence of artifacts such as muscular contractions. For rejection of ocular and cardiac artifacts data were submitted to extended infomax independent component analysis (ICA)^41^. Briefly, ICA returns a set of spatial filters, which, when matrix-multiplied with the data, yield component activations that are maximally temporally independent from each other. By visual inspection of component maps and component time courses, we identified those independent components reflecting eye blinks or movements and ECG artifacts^42,43^. Back-projection of the remaining non-artifactual components revealed corrected EEG data. Finally, data were re-referenced to common average.

### Spectral Analysis

Frequencies up to 40 Hz were analyzed using a sliding Hanning-window Fourier transformation with a window length of 500 ms and a step-size of 10 ms. For the analysis of frequencies higher than 40 Hz spectral analyses of the EEG data were performed using a sliding window multi-taper analysis^44^. In short, the data were multiplied by N > 1 orthogonal tapers and Fourier transformed, and the N spectral estimates are finally averaged. In case of power estimation, the spectra for each individual taper were magnitude squared after Fourier transformation. As data tapers, we used the leading 2TW-1 prolate spheroidal (slepian) sequences, where T denotes the length of the tapers and W the half bandwidth. These tapers optimally concentrate the spectral energy of the signal over the desired half-bandwidth W. Averaging across trials was finally performed in the frequency domain. A window of 500 ms length was shifted over the data with a step size of 10 ms. Spectral smoothing of 10 Hz was achieved by 3 slepian tapers. Time-frequency results were expressed as percent signal change relative to baseline, using a baseline interval from −1000 ms to 0 ms prior to stimulus onset. The gamma band was defined as frequencies between 40 to 100 Hz.

### Source Localization

For each subject, data sets were averaged for each block. Source reconstruction was applied using exact low resolution brain electromagnetic tomography (eLORETA). A realistic 3-shell model was constructed of the Montreal Neurological Institute template brain (MNI; http://www.mni.mcgill.ca). Using this head model and a lead field tensor for approximately 10.000 source points, an eLORETA spatial filter was constructed^45^ for each grid point. The eLORETA filter is linear and non-adaptive. Applying this filter, which consists for each grid point of an Nx3 matrix for N sensors and 3 dipole directions, on data is a weighted minimum norm inverse solution with weights designed such that, for a single dipolar source, the reconstructed distributed source has the maximum amplitude at the location of the dipole. For statistical analysis of neuronal activity, a paired t-test was performed on the result for each grid point to estimate the signal change of each condition versus baseline. Subsequently t-values were transformed to z-scores. All results were corrected for multiple comparisons using a cluster randomization approach^46^.

### Connectivity analysis

To analyze neuronal coupling related to pain processing, we applied a multivariate interaction measure (MIM) approach^45,47^. The connectivity analysis was data driven and only nodes obtained from the source reconstruction results were fed into the analysis of MIM. Hence, we only calculated MIM values between the four sources obtained from the source analysis. MIM is a multivariate extension of the imaginary part of coherence: it is a multivariate linear measure of functional connectivity at a specified frequency robust to artifacts resulting from volume conduction, implying that a non-vanishing value cannot be explained by an instantaneous mixture of sources that are actually independent. This is in contrast to classical coherence and also to other newly developed coherence based methods like icoh^48,49^ which is in particular designed as a measure of directed (and direct) interaction. For MIM, for each pair of grid points the functional dependence is measured between the two 3-dimensional spaces spanned by three possible dipole orientations for each grid point. This measure is in contrast to bivariate measures for which at first source orientation are estimated by maximizing the power which may lead to missing relevant interactions. For statistical analysis of neuronal communication, a paired t-test was performed for each connection to estimate the signal change of each condition versus baseline.

### Statistical analysis

For statistical analysis, the statistics toolbox running under Matlab (The Mathworks, Natick, MA) and SPSS 20 (IBM, NY) was used. Because the gamma-band activation was most pronounced in the central region, all sensor level analyses were performed using the data of the centro-frontal region of interest. All variables were first tested with a one-sample Kolmogorov-Smirnov test for normal distribution. As no deviations from the normal distribution were detected, we used parametrical statistics. Pain ratings, heart rate variability parameters, gamma-power values and source reconstruction CSD values were analyzed using repeated measures 2-way analyses of variance (2 × 4 ANOVA) testing for the effects of treatment (sham versus verum) and repetition (Pre, Treat 1, Treat 2, Treat 3). Significant main and interaction effects were then subjected to post-hoc paired t-tests. For all analyses, the critical p-value was set to *P* < 0.05. The p-values were corrected after adjustment of the degree of freedom of all effects with more that 2 factor levels by multiplying with the Greenhouse-Geisser epsilon values. Source reconstruction results were corrected for multiple comparisons using a cluster randomization approach^46^.

## Electronic supplementary material

Supplementary Figure 1
