# Supplementary material for: Acupuncture analgesia involves modulation of pain-induced gamma oscillations and cortical network connectivity
Source: Sci Rep. 2017 Nov 24;7:16307. doi: 10.1038/s41598-017-13633-4 (PMC5701238; doi:10.1038/s41598-017-13633-4)
Supplement: Supplementary file 1 — Supplementary Figure 1 [file 41598_2017_13633_MOESM1_ESM.pdf]

Supplementary Information

Title: Acupuncture analgesia involves modulation of pain-induced gamma oscillations and cortical network connectivity

Michael Hauck, Sven Schröder, Gesa Meyer-Hamme, Jürgen Lorenz, Sunja Friedrichs, Guido Nolte, Christian Gerloff, Andreas K Engel

Supplementary Figure 1

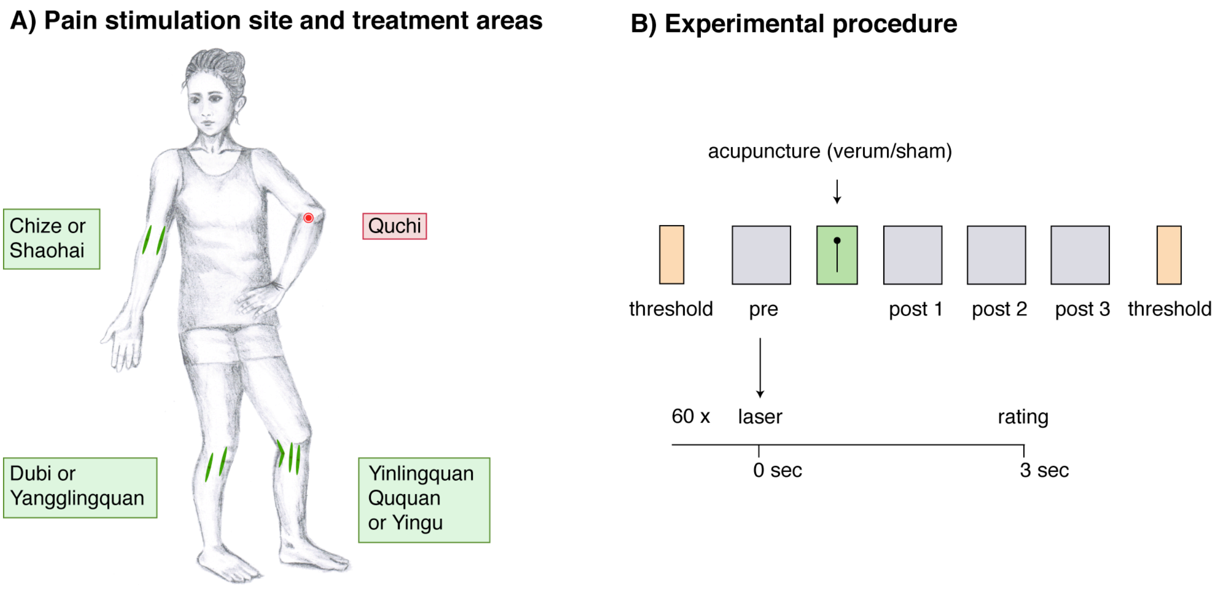

A) The pain stimulation side was located in a circular area (radius 2 cm) close to acupoint Quchi (LI11), at the left elbow, as marked by the red circle. Treatment was applied to reflex areas on distant corresponding body regions around points Chize (LU5) or Shaohai (HT3) at the right arm, Dubi (ST35) or Yanglingquan (GB34) at the right leg and Yinlingquan (SP9), Ququan (LIV8) or Yingu (KID10) as marked by the green shadings at the figure. The pattern of treatment was selected by a systematic physical examination for pain sensitive reflex areas (Ashi) in these regions immediately beforehand the application of sham or verum plasters.

B) The experimental procedure was identical for the two experimental days (verum and sham acupuncture). Pain thresholds were collected before and after the experimental blocks. The experiment started with a pre-block consisting 60 laser stimuli. Three seconds after each laser stimulus the subjects had to verbally rate the pain. This block was followed by the acupuncture or sham treatment intervention. After the intervention, three further blocks with 60 laser stimuli were applied in identical manner (post 1 – post 3).
